# Brief Communication: Sexual dimorphic expression of myostatin and follistatin like-3 in a rat trans-generational under-nutrition model

**DOI:** 10.1186/1743-7075-7-44

**Published:** 2010-05-20

**Authors:** Hassendrini N Peiris, Anna P Ponnampalam, Murray D Mitchell, Mark P Green

**Affiliations:** 1 The Liggins Institute, and National Research Centre for Growth and Development, University of Auckland, 2-6 Park Avenue, Private Bag 92019, Auckland, New Zealand; 2 Current Address: University of Queensland Centre for Clinical Research, RBWH Campus, Herston, Brisbane Qld 4029 Australia

## Abstract

The detrimental effects of maternal under-nutrition during gestation on fetal development are well known with an increased propensity of metabolic disorders identified in the adult offspring. Understanding exactly how and by which molecular pathways inadequate nutrition can impact upon offspring phenotype is critical and necessary for the development of treatment methods and ultimately prevention of any negative health effects. Myostatin, a negative regulator of muscle development, has recently been shown to effect glucose homeostasis and fat deposition. The involvement of myostatin in glucose metabolism and adipogenesis thus supports its ability to act in the continued alterations to the postnatal phenotype of the offspring. This hypothesis was examined in the current study using a trans-generational gestationally under-nourished rat model exposed to a high-fat (HF) diet post-weaning. The body weight, body fat, plasma glucose and insulin concentrations of the offspring, both male and female, were investigated in relation to the protein expression of myostatin and its main inhibitor; follistatin like-3 (FSTL-3), in skeletal muscle of mature offspring. Sexual dimorphism was clearly evident in the majority of these measures, including myostatin and FSTL-3 expression. Generally males displayed higher (*P < 0.05*) myostatin precursor and dimer expression than females, which was especially apparent (*P < 0.01*) in both chow and HF trans-generationally undernourished (UNAD) groups. In females only, myostatin precursor and dimer expression was altered by both trans-generational under-nutrition and postnatal diet. Overall FSTL-3 expression did not differ between sexes, although difference between sexes within certain treatments and diets were evident. Most notably, HF fed UNAD females had higher *(P < 0.05) *FSTL-3 expression than HF fed UNAD males. The former group also displayed higher *(P < 0.01) *FSTL-3 expression compared to all other female groups. In summary, myostatin may prove to be a key mediator of the effects of inadequate prenatal nutrition, independently or in combination with a high-fat postnatal diet on offspring phenotype. Consequently, further study of myostatin may provide a novel therapeutic pathway for the treatment of metabolic disorders; however, it is vital that the influence of nutrition and gender should be taken into consideration.

## Introduction

Myostatin initially designated as growth differentiation factor 8 (GDF-8), is a distinctive member of the TGF-β super-family, retaining many of the characteristic features found in this family [1]. The functional importance of myostatin is inferred by the high level of sequence homology and conservation seen across a number of species. Synthesized as a precursor protein, myostatin is proteolytically cleaved twice to release the biologically mature form of myostatin, with dimerisation of the mature protein creating the active form of myostatin [1]. The binding of the myostatin dimer to its receptor (ActRIIB) initiates the Smad mediated signaling pathway, which results in the transcription and expression of genes needed to mediate the negative regulation of muscle development. Myostatin function is controlled by a number of inhibitors, the most potent being follistatin like-3 (FSTL-3) [2].

Myostatin was initially identified as a negative regulator of muscle development where its inactivation in mice resulted in offspring with a two to three-fold increase in muscle mass [3]. Subsequently, myostatin was identified to affect glucose uptake in the human placenta [4]. Recently, altered placenta myostatin concentrations were identified in developmentally programmed rat model that supports a role as a mediator of this phenomenon [5]. Myostatin has also been shown to be crucial in the modulation of glucose homeostasis and adipogenesis [6,7]. The aim of this study was to utilise a model of trans-generational maternal under-nutrition to investigate potential sexual dimorphic changes in the expression of myostatin and FSTL-3 in rat skeletal muscle.

## Materials and methods

**Figure 1:**
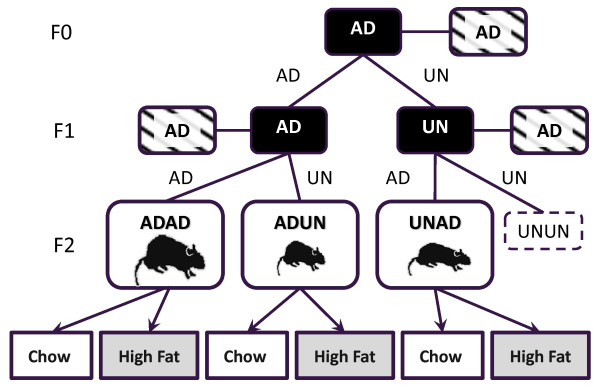
**The experimental design employed in this study**. Gestational diets are represented by AD = *ad libitum *and UN = 30% of the *ad libitum *diet. Hatched boxes are males and in black are the females. The relative difference in body size between F2 generation males and females is represented diagrammatically.

The animal model depicted (Figure 1) was based upon an established model of under-nutrition [8-10]. Briefly, virgin female Wistar rats (F0) reared on an *ad libitum *(AD) standard chow diet (2018 Teklad Global Rodent Diet; Bicester, UK) were mated at D120 ± 5 of age with males also fed an AD standard chow diet. Following confirmation of mating, the females were individually housed and received either an AD (AD group) or 30% of the AD chow diet (UN group) throughout gestation. During lactation dams of both groups were on an AD diet. Litter size was standardized on D1 to 10 pups per litter to standardize nutrition until weaning. Female offspring from these pregnancies (F1) were weaned and fed chow AD until D120 ± 5 of age before being mated with AD males and fed either AD or UN during gestation. Resulting in three groups ADAD, ADUN and UNAD; UNUN animals were not bred for ethical reasons.

F1 females litter size was standardized on D1 to 10 pups, 5 males and 5 females where possible. All these F2 pups were nursed by their own dams that were fed AD *until *weaning (D22). At weaning half the offspring from each of the three groups were fed either a chow or high fat (HF, 60% kcal as fat; D12492 Research Diets Inc, NJ, USA) diet *ad libitum *until being euthanized at D140. The body weights of F2 animals were recorded from birth until D140. On D130, body measurements and composition were determined in F2 animals (n = 10 per sex per diet per group) by DEXA using a LUNAR Prodigy Scanner (GE Medical Systems, WI, USA). On D140 after an overnight fast, the animals were euthanised via decapitation under sodium pentobarbital anesthesia (60 mg kg, I.P.). Blood was collected, centrifuged for 15 mins at 1500 × *g *at 4°C and stored at -20°C for further analysis. Skeletal muscle samples were collected from the left hind leg quadriceps, snap frozen and stored at -80°C. All animal procedures undertaken were approved by the University of Auckland Animal Ethics Committee.

Plasma glucose was measured along with quality controls using commercial kits (Roche Diagnostics Corp.; IN, USA) on an auto-analyzer (Roche/Hitachi 9002 Analyzer; Hitachi, Tokyo, Japan). Plasma insulin was determined by a rat insulin ELISA (Mercodia; Uppsala, Sweden) following the manufacturers' instructions. The insulin assay sensitivity was 0.07 μg/l and the intra- and inter-assay coefficients of variation (CVs) were 3.4% and 6.3%.

### Protein extraction and Western blotting

Proteins were extracted from male and female D140 muscle samples and Western blot analysis performed [11,12]. Antibodies from Santa Cruz Biotechnology (CA, USA) were used for the detection of myostatin (1:3000 dilution; Goat Polyclonal GDF-8 (F-13), Sc-34781) and FSTL-3 (1:5000 dilution; Goat Polyclonal FLRG (N-18), Sc-21302) in conjunction with a secondary Polyclonal Rabbit Anti IgG/HRP antibody (1:10000 dilution). The relative optical densities were normalized to sheep muscle expression of myostatin and FSTL-3 protein.

### Statistical analysis

All analyses were run using a general linear mixed model followed by Tukey's post hoc comparison performed on SAS software version 9.1 (SAS institute, Cavy, NC). Differences in bodyweight and composition, plasma metabolite concentrations and the relative expression of myostatin and FSTL-3 in skeletal muscle were analysed with group, sex and diet included in the model, and all interactions between the main effects were analysed. All data presented are as means ± SEM.

## Results

Comparisons of D130 body composition are shown in Table 1 and Table 2. Male offspring were heavier *(P < 0.0001) *than females (Table 1). Males and females were heavier *(P < 0.05) *on an HF diet compared to their chow fed contemporaries; the ADAD group was the heaviest within HF fed animals *(P < 0.01)*. A trend toward a higher percentage of body fat in the males compared to females was observed with significance reached in the ADUN chow and HF fed offspring *(P < 0.05)*. HF fed offspring had an elevated body fat percentage but lower lean body percentage compared to chow-fed offspring. Interestingly, both UNAD HF fed males and females had a lower *(P < 0.01) *body fat percentage compared to ADAD contemporaries (Table 1). Differences in plasma glucose and insulin concentrations were also evident between sexes and groups (Table 2). Most strikingly an elevated glucose concentration was seen in UNAD HF females (not males) compared to all other groups.

**Table 1:** Mature female and male body weight, body fat percentage and lean body percentage.

	Body weight		Body fat %		Lean Body %	
	Female	Male	Female	Male	Female	Male
	
	***Mean ± SEM***	***Mean ± SEM***	***Mean ± SEM***	***Mean ± SEM***	***Mean ± SEM***	***Mean ± SEM***
**Chow fed**						
ADAD	323.9 ± 10.4^**ax**^	564.6 ± 13.5^**ay**^	25.9 ± 2.2^**ax**^	27.0 ± 2.3^**acx**^	66.23 ± 2.93^**ax**^	66.64 ± 1.91^**ax**^
ADUN	273.5 ± 11.2^**bx**^	528.3 ± 12.2^**by**^	17.7 ± 2.0^**bx**^	24.9 ± 2.2^**acy**^	73.68 ± 0.61^**bx**^	69.38 ± 1.61^**abx**^
UNAD	305.5 ± 10.8^**adx**^	552.9 ± 8.4^**aby**^	23.4 ± 2.2^**abx**^	25.9 ± 2.2^**acx**^	67.70 ± 1.99^**abx**^	67.04 ± 1.57^**adx**^
						
**High fat fed**						
ADAD	365.8 ± 10.4^**cx**^	692.5 ± 39.1^**cy**^	39.1 ± 2.2^**cx**^	40.9 ± 2.3^**bx**^	53.42 ± 3.79^**bcx**^	52.73 ± 1.62^**bx**^
ADUN	308.7 ± 11.7^**bdx**^	621.6 ± 11.7^**dy**^	27.0 ± 2.2^**adx**^	41.4 ± 2.2^**by**^	64.63 ± 1.86^**ax**^	52.10 ± 1.21^**cy**^
UNAD	335.6 ± 10.8^**adx**^	636.7 ± 30.8^**ey**^	30.8 ± 2.2^**adx**^	32.5 ± 2.2^**cx**^	61.16 ± 2.00^**adx**^	61.68 ± 3.83^**adx**^

Body weight: Female ADAD chow (n = 15), ADUN chow (n = 13), UNAD chow (n = 14), ADAD high fat (n = 15), ADUN high fat (n = 12), UNAD high fat (n = 14); Male ADAD chow (n = 9), ADUN chow (n = 11), UNAD chow (n = 23), ADAD high fat (n = 9), ADUN high fat (n = 12), UNAD high fat (n = 22). Body fat and lean muscle percentage: Female ADAD chow (n = 10), ADUN chow (n = 9), UNAD chow (n = 10), ADAD high fat (n = 10), ADUN high fat (n = 10), UNAD high fat (n = 10); Male ADAD chow (n = 9), ADUN chow (n = 10), UNAD chow (n = 10), ADAD high fat (n = 10), ADUN high fat (n = 10), UNAD high fat (n = 10). Significant differences (P < 0.05) between the experimental groups are shown by letters **a **to **e **(read down column). Significant differences (P < 0.05) between genders shown by letters **x **and **y **(read across column).

**Table 2:** Mature female and male plasma insulin and glucose concentrations.

	Insulin (μg/l)		Glucose (mmol/l)	
	Female	Male	Female	Male
	
	***Mean ± SEM***	***Mean ± SEM***	***Mean ± SEM***	***Mean ± SEM***
**Chow fed**				
ADAD	2.2 ± 0.4^**abx**^	2.0 ± 0.6^**ax**^	8.6 ± 0.3^**ax**^	8.5 ± 0.4^**abx**^
ADUN	1.5 ± 0.4^**ax**^	3.5 ± 0.5^**bcy**^	8.3 ± 0.3^**ax**^	8.8 ± 0.4^**abx**^
UNAD	1.6 ± 0.4^**ax**^	2.5 ± 0.3^**aby**^	9.1 ± 0.3^**ax**^	8.1 ± 0.2^**ay**^
				
**High fat fed**				
ADAD	2.9 ± 0.4^**bcx**^	4.0 ± 0.6^**bcx**^	8.7 ± 0.3^**ax**^	9.1 ± 0.4^**bx**^
ADUN	3.1 ± 0.5^**bcx**^	3.1 ± 0.5^**abcx**^	8.9 ± 0.3^**ax**^	8.1 ± 0.3^**ax**^
UNAD	3.6 ± 0.4^**cx**^	4.1 ± 0.3^**bcx**^	10.2 ± 0.3^**bx**^	9.4 b ± 0.2^**by**^

Insulin and glucose: Female ADAD chow (n = 9), ADUN chow (n = 7), UNAD chow (n = 6), ADAD high fat (n = 9), ADUN high fat (n = 6), UNAD high fat (n = 8); Male ADAD chow (n = 4), ADUN chow (n = 5), UNAD chow (n = 15), ADAD high fat (n = 4), ADUN high fat (n = 6), UNAD high fat (n = 14). Significant differences (P < 0.05) between the experimental groups are shown by letters **a **to **e **(read down column). Significant differences (P < 0.05) between genders shown by letters **x **and **y **(read across column).

**Figure 2:**
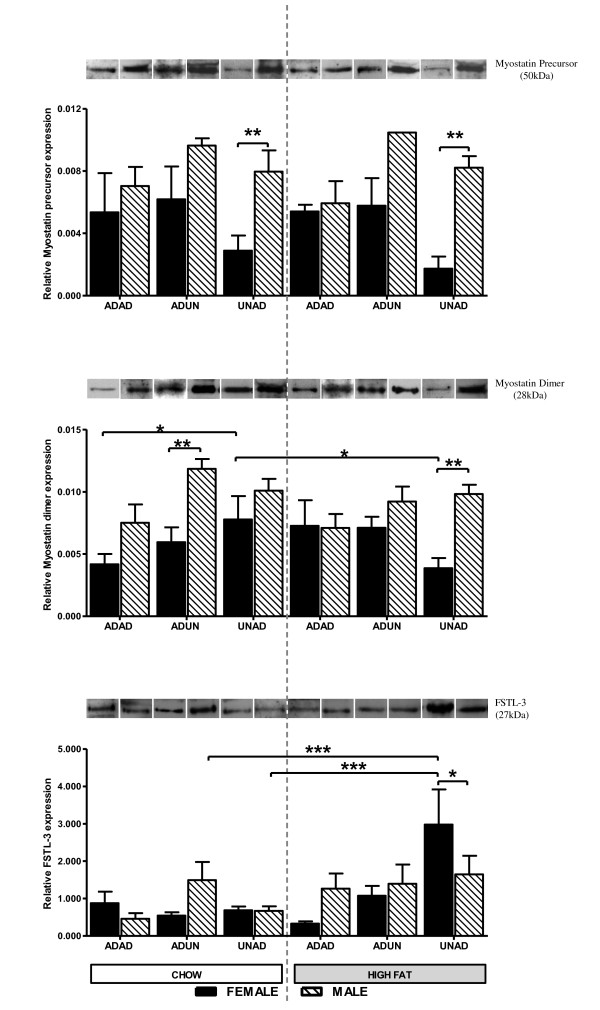
**Myostatin precursor, dimer and FSTL-3 expression**. **(a) **D140 female and male skeletal muscle tissue expression of myostatin precursor (50 kDa). Female ADAD chow (n = 3), ADUN chow (n = 4), UNAD chow (n = 7), ADAD high fat (n = 4), ADUN high fat (n = 4), UNAD high fat (n = 3); Male ADAD chow (n = 4), ADUN chow (n = 3), UNAD chow (n = 4), ADAD high fat (n = 2), ADUN high fat (n = 1), UNAD high fat (n = 5). **(b) **D140 female and male skeletal muscle tissue expression of myostatin dimer (28 kDa). Female ADAD chow (n = 6), ADUN chow (n = 7), UNAD chow (n = 7), ADAD high fat (n = 5), ADUN high fat (n = 6), UNAD high fat (n = 6); Male ADAD chow (n = 4), ADUN chow (n = 3), UNAD chow (n = 5), ADAD high fat (n = 4), ADUN high fat (n = 3), UNAD high fat (n = 5). **(c) **D140 female and male skeletal muscle tissue expression of FSTL-3 (27 kDa). Female ADAD chow (n = 3), ADUN chow (n = 6), UNAD chow (n = 7), ADAD high fat (n = 5), ADUN high fat (n = 6), UNAD high fat (n = 5); Male ADAD chow (n = 4), ADUN chow (n = 3), UNAD chow (n = 4), ADAD high fat (n = 3), ADUN high fat (n = 3), UNAD high fat (n = 5). Females are represented by black bars and males by the hatched bars. The graphs are divided on either side of the dashed line by postnatal diet (chow or high fat fed) of offspring. Gestational diet is on the x-axis and relative expression of the proteins on the y-axis. Data given as mean optical densities relative to positive control, sheep muscle ± SEM. Significance is shown by the stars where P < 0.05 *, P < 0.01 **, P < 0.001***.

Generally males displayed higher (*P < 0.05*) precursor (Figure 2a) and dimer (Figure 2b) expression than females, which was especially apparent (*P < 0.01*) in both chow and HF UNAD, and to a lesser extent (*P < 0.05*) in chow-fed ADUN animals. In females only, myostatin precursor and dimer expression was altered by both trans-generational under-nutrition and postnatal diet, demonstrated by the UNAD females having a lower *(P < 0.05) *expression than ADAD and ADUN contemporaries and the dimer expression of UNAD HF-fed females being lower (P < 0.05) than UNAD chow-fed females. Overall FSTL-3 expression did not differ between sexes, although difference between sexes within certain treatments and diets were evident (Figure 2c). Most notably, UNAD HF females had higher *(P < 0.05) *FSTL-3 expression than UNAD HF males and in fact, higher *(P < 0.01) *FSTL-3 expression compared to all other female groups.

## Discussion

Sexually dimorphic differences were evident in the phenotypic measures investigated and notably, the skeletal muscle expression of myostatin and FSTL-3 proteins. The majority of these measures were also impacted upon by gestational and trans-generational under-nutrition and postnatal diet.

Generally higher myostatin precursor and dimer expression was identified in the males compared to females, whereas a consistent dimorphic difference in FSTL-3 expression was not evident; with only a few specific groups within diet showing differences. The increased expression of myostatin precursor in skeletal muscle of males compared to females is in agreement with studies in mice [13,14]. In contrast however to our study, these studies in mice identified the myostatin dimer expression, unlike the precursor, to be higher in females, which is surprising since it would be expected that both precursor and dimer had increased expression in the same sex, as in the current study. Species specific differences in myostatin dimer expression and the use of different antibodies between the two studies in order to detect the dimer, and thus potentially different binding sites, may however explain the discrepancy.

In this study, irrespective of sex, the expression of FSTL-3, myostatin precursor and dimer were varied in the UNAD HF fed offspring. This matches the phenotypic data, as UNAD animals fed an HF diet post-weaning, irrespective of sex, were lighter and had a reduced percentage body fat than ADAD animals, as well as ADUN animals (males only). Recently, the finding that myostatin maintained adipocytes at an immature state and thus influenced fat mass [4], suggest myostatin can affect two different processes; fat deposition and glucose uptake. Phenotypic differences in body composition were also linked to changes in plasma metabolite concentrations, as plasma glucose and insulin concentrations were elevated in the UNAD group (mainly in females) demonstrating hyperglycaemia, compared to the two other groups. Higher blood glucose concentrations, as evident in diabetes, are known to lead to reduced body weight of offspring [15,16]. The increased plasma glucose concentrations evident in UNAD offspring could be influenced by the expression of myostatin and FSTL-3 in skeletal muscle, with limited fiber number and size potentially restricting uptake of glucose from the circulation, although further studies are needed to examine this hypothesis. If true, myostatin may be a possible mediator of offspring metabolic and physical phenotype.

## Conclusion

Myostatin and FSTL-3 are sexually dimorphic in their expression and both are also affected by gestational and trans-generational under-nutrition. Thus myostatin may be a key mediator of the effects of maternal under-nutrition; altering fetal development, the mature phenotype of offspring, and even the phenotype of subsequent generations. Further study of myostatin may provide a novel therapeutic pathway for the treatment of metabolic disorders; however, it is vital that the influence of nutrition and gender should be taken into consideration.

## Abbreviations

ACTRIIB: Activin receptor IIB; AD: *ad libitum*; ADAD: shows F0: fed AD diet during gestation (AD); F1: fed AD diet during gestation (AD); ADUN: shows F0: fed AD diet during gestation (AD); F1: fed 30% of AD diet during gestation (UN); D: Day followed by the number of days *e.g. *D140; DEXA: Dual Energy X-ray Absorptiometry; F0: Founder generation; F1: First generation; F2: Second generation; FSTL-3: Follistatin like-3; GDF-8: Growth differentiation factor 8; HF: High fat; kcals: Kilo calories; SMAD: Drosophila protein, combination of mothers against decapentaplegic (MAD) and the C. elegans protein (SMA); TGF-β: Transforming Growth Factor-beta; UN: 30% of the *ad libitum *diet during gestation; UNAD: shows F0: fed 30% AD diet during gestation (UN); F1: fed AD diet during gestation (AD).

## Competing interests

The authors declare that they have no competing interests.

## Authors' contributions and information

HNP carried out the protein extractions, Western blotting, data analysis and drafted the manuscript. APP aided with the experimental design, data interpretation and wrote the manuscript. MPG was responsible for the experimental design, undertaking of the animal study and plasma assays, data analysis and wrote the manuscript. MDM had overall supervision and gave final approval of the manuscript to be published. All authors have read and approved the final manuscript.
